# Effect of Content and Size of Reinforcements on the Grain Evolution of Graphene-Reinforced Aluminum Matrix Composites

**DOI:** 10.3390/nano11102550

**Published:** 2021-09-29

**Authors:** Qi Wu, Pengfei Cai, Lianchun Long

**Affiliations:** Faculty of Materials and Manufacturing, Beijing University of Technology, Beijing 100124, China; Caipf@emails.bjut.edu.cn

**Keywords:** graphene-reinforced aluminum, grain growth, heat treatment, Monte Carlo method

## Abstract

Graphene-reinforced aluminum matrix composites (GRAMCs) attract great interest in industries due to their high performance potential. High-temperature processes such as sintering and aging are usually applied during the preparation of GRAMCs, leading to grain coarsening that significantly influences its properties. In this work, a modified 3D Monte Carlo Potts model was proposed to investigate the effect of content and size of graphene on the grain evolution during the heat treatment of GRAMCs. Grain growth with graphene contents from 0.5 wt.% to 4.5 wt.% and sizes from 5 μm to 15 μm were simulated. The grain growth process, final grain size and morphology of the microstructure were predicted. The results indicated that both the content and size of the reinforcements had an impact on the grain evolution. The pinning effect of grain size can be enhanced by increasing the content and decreasing the size of graphene. Agglomeration and self-contacting phenomena of the graphene arose obviously when the contents and sizes were relatively high. The average grain size decreased by 48.77% when the content increased from 0.5 wt.% to 4.5 wt.%. The proposed method and predicted regulations can provide a reference for the design and fabrication of GRAMCs.

## 1. Introduction

Aluminum matrix composites (AMCs) have been applied in aerospace, construction, transportation and other fields because of their outstanding performance.

With the increasing demand for performance improvements, it is important to seek new reinforcements to fabricate more advanced AMCs. Compared to traditional reinforcing phases such as ceramics, oxides and metals, graphene has better mechanical and physical properties [1]. Therefore, preparing new AMCs by using graphene as a reinforcement has great application potential [2]. Graphene nanoplatelets (GNPs) are easier to generate and more stable than single-layer graphene, so they are widely used as a reinforcing phase to fabricate graphene-related materials and reinforce aluminum matrix composites such as GNPs/Al [3].

Common fabrication methods of GRAMCs include powder metallurgy, casting, severe plastic deformation, additive manufacturing and so on [4]. Latief et al. [5] prepared GNPs/Al composites with a mass ratio of up to 5 wt.% by powder metallurgy and hot extrusion methods. The performance tests showed that increasing the sintering temperature can improve the forming efficiency; however, this affected the grain morphology and reduced the mechanical properties. Bartolucci et al. [6] also reported that excessive time of high-temperature heat treatment reduced the mechanical properties of GRAMCs. The experimental observations from Khodabakhshi et al. [7] showed that the extent of grain refinement of aluminum matrix containing GNPs was more significant compared with pure aluminum or aluminum alloys after the same thermal–mechanical processing. Brodova et al. [8] and Xie et al. [9] proposed equal channel angular pressing (ECAP) and friction stir processing (FSP) methods to prepare GNP/Al composites, respectively. The severe deformation process refined the grain size and improved the strength and toughness of the obtained composites. Pérez-Bustamante et al. [10] studied the effects of the parameters of high-energy ball milling and sintering on the properties of the final composites. The results established that different process parameters had a great impact on the grain size and morphology of GNPs/Al; hence, there was potential for optimization.

In addition to the parameters of the preparation process, such as mechanical and thermal conditions, the reinforcing phase is an important factor that affects the final properties. The content of the reinforcements (volume fraction or mass ratio) is undoubtedly one of the most important parameters. Besides content, the size, stacking orientation and spatial distribution of graphene have all been proven to have a certain impact on the final properties of GRAMCs in recent years [11]. The grain size of the matrix has a decisive influence on the mechanical strength, ductility, hardness, corrosion resistance and fatigue properties of GRAMCs. Inhibiting the grain coarsening of the matrix is one of the most effective ways to improve the properties of GRAMCs. Therefore, it is of great significance to study the grain size evolution during the preparation of GRAMCs, particularly with heat treatments. However, the combinations of parameters of reinforcements and preparation processes are very large and complex. Using experimental methods to study the influence of the above factors on grain evolution is costly, and it is quite difficult to investigate the dynamic evolution process.

Numerical simulation has evident advantages in studying the grain evolution of polycrystalline materials under thermal conditions. First principle (FP), molecular dynamics (MDs), phase field (PF), Monte Carlo (MC) and Cellular Automata (CA) methods have all been applied to the study of material microstructure evolution at different scales. The grain growth behavior of AMCs is usually between 10^−1^ μm and 10^2^ μm. For this scale, MC and CA methods have advantages such as high computational efficiency, scale applicability and experimental verifiability. Therefore, MC and CA methods have been rapidly applied in the fields of grain evolution, property prediction, molten pool solidification, abnormal growth and pinning phenomenon in numerical investigations [12,13,14,15,16]. Soucail et al. [17] used a 2D-MC model to study the grain growth process of AMCs containing granular reinforcements, and showed that the MC method can properly simulate the grain growth regulations under the condition of large-span volume fraction of reinforcements, even from 0.01 vt.% to 10 vt.%. Gao et al. [18] predicted the limit grain size of particle-reinforced material by a 2D hexagonal-CA model. Li et al. [19,20] proposed an empirical grain growth formula method for materials with particle reinforcements higher than 1 vt.% by a 2D square-CA model. Han et al. [21] reported the relationship between grain size and particle size and content, and proposed an empirical relationship via a 2D-CA model. Chang et al. [22,23] and Du et al. [24] both studied the influence of the shapes of reinforcements on the grain evolution of composites. Their results indicated that the pinning effect changed with the changing of reinforcement parameters.

Compared with 2D models, 3D models are able to simulate many more directions of grain growth, and as a result represent richer morphologies of grains and reinforcing phases. Therefore, the 3D model is considered to be more accurate in predicting the grain growth process of composites, but is accompanied by the rapid increase in calculation scale and the complexity of algorithms. Graphene is 2D lamellar nano-reinforcement, so the actual grain growth process of GRAMCs can only be predicted properly in the 3D model. However, until now, there have been few reports on the prediction model of grain evolution of metal matrix composites containing nano-lamellar reinforcing phases, which greatly limits the design and development of GRAMCs. In this work, a modified 3D Monte Carlo Potts (MCP) model was proposed to simulate the grain evolution with various parameters of reinforcements under heat treatment. Grain growth of GRAMCs with graphene contents from 0.5 wt.% to 4.5 wt.% and sizes from 5 μm to 15 μm were simulated. The grain growth process, final grain size and morphology of the microstructure were also predicted and further analyzed. This method and obtained results provide a reference for the design and fabrication of GRAMCs.

## 2. Description of Model

A lattice system with a total number of *N*_mnp_ = *m* × *n* × *p* cells was adopted to represent the actual 3D spatial region of GRAMCs. Cells were all regular cubes, each cell contacts with 26 neighboring cells. An orientation degree was assigned to every cell. Adjacent cells with the same orientation degree formed the same grain, otherwise forming the grain boundary. The size of each grain *D* was determined by the mean value of the major and minor axes of the region surrounded by the cells constituting the same grain. The orientation degrees of the matrix were assigned from positive integers 1 to *q*, and the orientation degree of reinforcements was fixed at −1. Spatial locations and surface orientations of graphene were generated randomly. At the beginning time of simulation, the orientation degrees of matrix were also generated randomly. The driving force of grain growth was considered as grain boundary migration. The influence of heat treatment was then modeled by the energy criterion according to the principle that grain boundary energy tends to decrease. According to the energy criterion for each lattice cell, its free energy *E*_i_ is calculated by Hamilton formula,

(1) $$E_{i}=J_{i}\sum_{1}^{26}\left(1-\delta_{\mathrm{ij}}\right)$$

where *J*_i_ is a function representing unit grain boundary energy at position i, *δ* is the Kronecker function, if grain orientation degree at position i is equal to that at position j, then the value of *δ*_ij_ equals 1, otherwise the value will be 0. By using Equation (1), the total energy of the system *E*_1_ can be calculated from the summation of all cells. For a certain central lattice cell, if the grain orientation degree transforms to a randomly selected neighbor cell, then the new total energy is denoted by *E*_2_. The change in total energy can be then calculated by,

(2) $$\Delta E=E_{2}-E_{1}$$

then the probability *p* for whether to accept the above grain orientation degree transformation is determined by,

(3) $$p=\left\{ \begin{array}{ll} 1, & \Delta E\leq 0\\ e^{-\frac{\Delta E}{k_{B}T_{\mathrm{abs}}}}, & \Delta E>0 \end{array}\right.$$

where *k*_B_ is the Boltzmann constant, *T*_abs_ is the absolute temperature. In this model, the value of term *J*_i_/*k*_B_*T*_abs_ was assumed to be 1 during the numerical calculations [25].

The MCP model controlled the simulation time through the number of iteration steps—*MCS*. In every *MCS*, the total number of *N*_mnp_ lattice cells was randomly selected to judge the above energy criterion in sequence, and in this way the model simulated grain evolution. The model time *MCS* needed to be correlated to the actual time and temperature history of the material. In this work, the model in [26] has been adopted. Migration velocity of grain boundaries during grain growth can be calculated by,

(4) $$v=\frac{AZV_{m}^{2}}{N_{a}^{2}h}\exp(\frac{\Delta S_{f}}{R})\exp(-\frac{Q}{RT})(\frac{2\gamma }{D})$$

where *A*, *Z*, *V*_m_, *R*, *N*_a_ and *h* are physical constants, *S*_f_, *Q* and *γ* are parameters of the matrix material. Grain growth kinetics predicted by MCP model can be fitted by,

(5) $$D=K_{1}\lambda {\left(MCS\right)}^{n_{1}}$$

where *K*_1_ is the model constant related to the slope of the grain growth curves, *n*_1_ is the model constant decided by the grain growth exponent, *K*_1_ and *n*_1_ are fitted as 1.01 and 0.43, respectively. *λ* is the actual size of the lattice cell. Velocity of grain boundary migration can be associated with average grain size by,

(6) v=dD/dt

then the calculation formula of the relationship between *MCS* and heat treatment parameters can be obtained by synthesizing Equations (4)–(6),

(7) $${\left(MCS\right)}^{2n_{1}}=\frac{4\gamma AZV_{m}^{2}}{N_{a}^{2}hK_{1}^{2}\lambda^{2}}\exp(\frac{\Delta S_{f}}{R})\sum [\exp(-\frac{Q}{RT_{i}})t_{i}]+{\left(\frac{D_{0}}{K_{1}\lambda }\right)}^{2}$$

where *D*_0_ is the initial grain size, determined by the randomly assigned orientation degrees at initial time. *t*_i_ and *T*_i_ represent that the thermal history has been divided into a series of time intervals for calculating purpose, *T*_i_ is the temperature in time interval *t*_i_. The details of the above parameters are listed in Table 1 [27].

**Figure 1:**
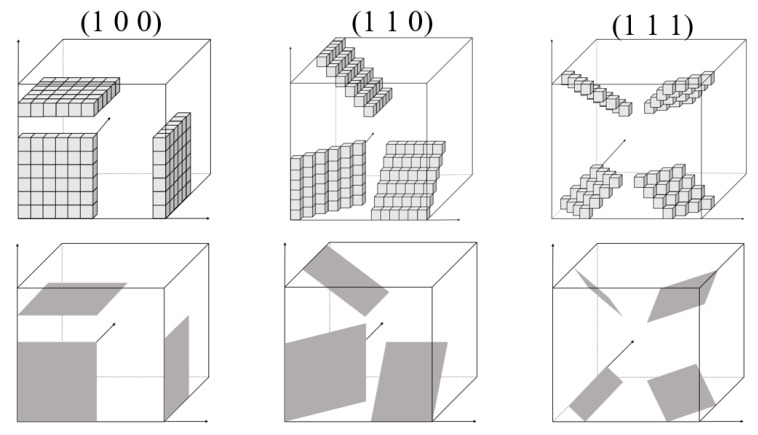
Modeled surface orientations of graphene.

The following assumptions and simplifications were introduced during the simulation:

(1). Ignoring the influence of initial microstructure by randomly assigning grain orientation degrees to lattice cells;
(2). The thickness of graphene was modeled to occupy one-cell size, to adapt to the huge scale differences in the thickness and diameter of graphene as a 2D-nanomaterial. The mass ratio of reinforcement was then calculated by the volume ratio and relative atomic mass;
(3). Periodical conditions were applied at the boundaries;
(4). Due to regular hexahedral lattice grids, graphene was idealize-modeled as groups of surfaces with the orientations of (1 0 0), (1 1 0) and (1 1 1), as shown in Figure 1.

**Figure 2:**
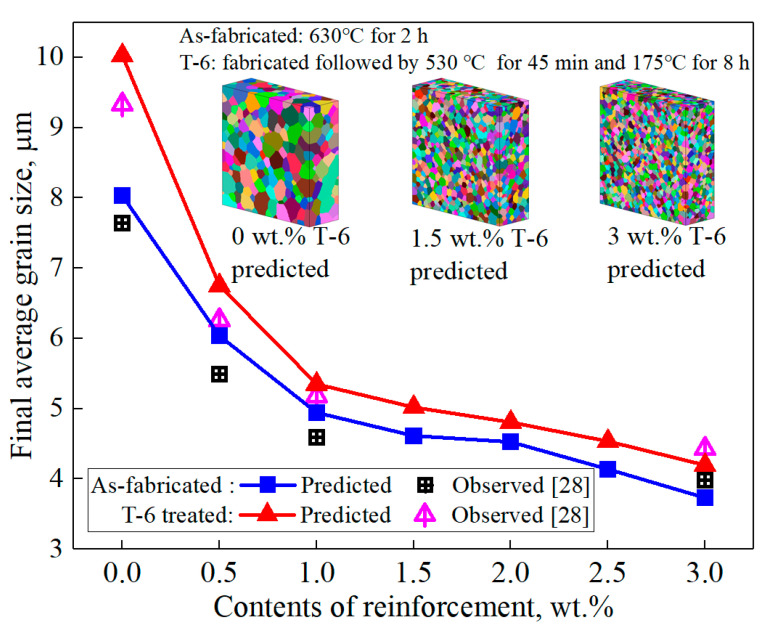
Validation of predicted results versus observations in the literature.

The experimental observations of grain growth of GRAMCs in the literature [28] were selected to validate the accuracy of the proposed model in this work. A simulation model that is completely consistent with the experimental parameters was established. The modeled size of graphene was 5 μm, and the contents were from 0 to the maximum value of 3 wt.%. The grid dimensions adopted were 300 × 300 × 100, *q* and *λ* were 100 and 0.5 μm, respectively. *MCS* calculated according to Equation (7) were 1124 for the as-fabricated state and 1575 for the T-6 heat treatment state, respectively. Thermal conditions, predicted results and comparisons are shown in Figure 2.

Comparing the predicted and observed values, the predicted grain size was a little higher than the experimental ones at low contents of graphene, except at 3 wt.%. This is because the pinning effect was relatively low in the model under the conditions of low contents, resulting in higher predicted grain growth rates. When the content was high, the spatial distribution density of graphene increased rapidly in the grid system, and the simulated pinning effect became obviously significant, resulting in a lower grain growth rate. Overall, the predicted final average grain size, changing trends and grain morphology were in good agreement with the experimental results in the literature. The comparison results prove the accuracy of the model.

## 3. Results and Discussion

Based on the validated MCP model, the effect of content and size of graphene on the grain evolution during heat treatment were further investigated. The simulated sizes of graphene ranged from 5 μm to 15 μm, and the contents ranged from 0.5 wt.% to 4.5 wt.%. According to previous works [27], the cost of computation can be reduced by reducing the number of grids along one specific direction, without influencing the simulation of the 3D grain evolution process. Therefore, a 300 × 300 × 50 grid was used, *q* and *λ* were chosen as 100 and 1 μm, respectively. Modeled thermal treatment was 600 °C kept for 6 h, which was within the commonly used ranges of powder metallurgy and provides sufficient time for grain growth [3,4]. The *MCS* obtained by Equation (7) was 2826 for all cases due to the same thermal history.

**Figure 3:**
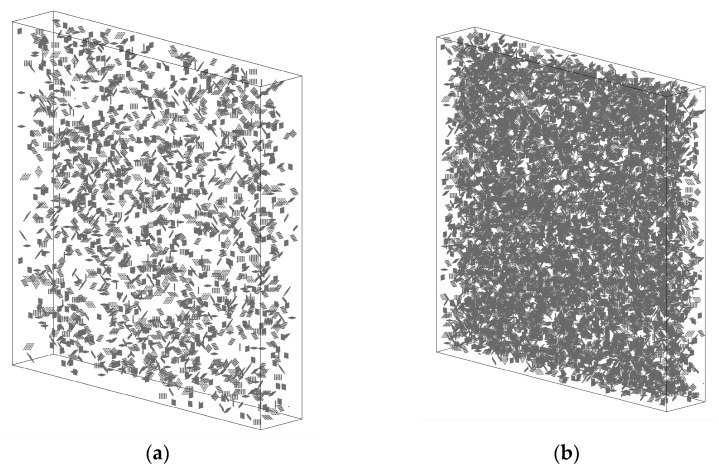

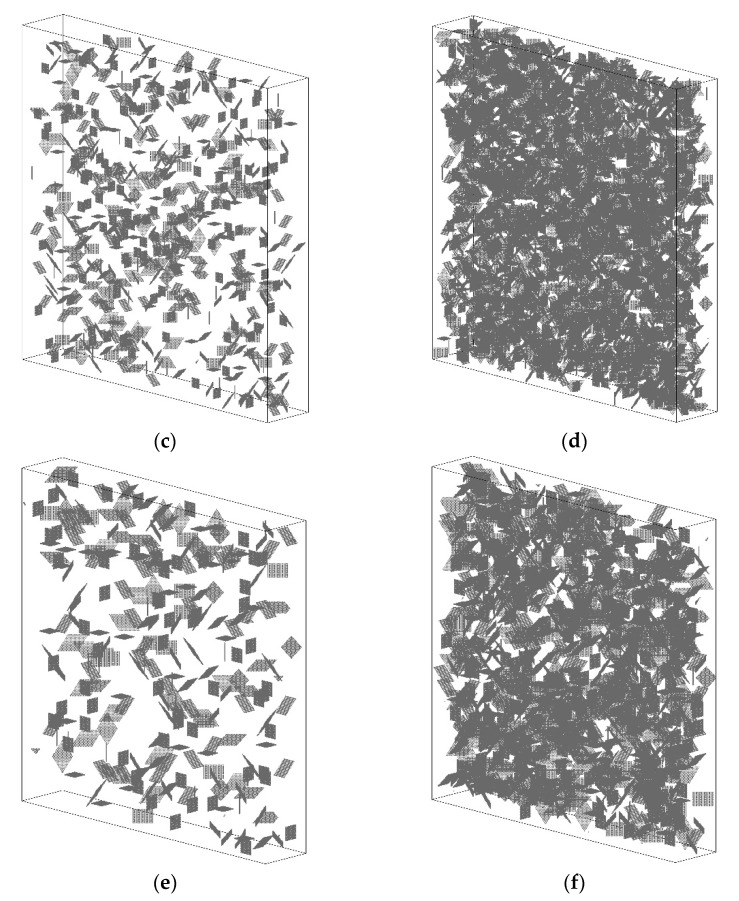
Spatial distribution of modeled graphene (**a**) 1 wt.% and 5 μm size, (**b**) 4 wt.% and 5 μm size, (**c**) 1 wt.% and 9 μm size, (**d**) 4 wt.% and 9 μm size, (**e**) 1 wt.% and 13 μm size, (**f**) 4 wt.% and 13 μm size.

The spatial distribution of several simulated parameters of graphene is shown in Figure 3, in which the locations of graphene are represented by gray points. It can be seen that when the size of graphene was small, its spatial distribution was very similar to the particle-reinforcing phase, and the influencing range of every single graphene was also small. With the increase in graphene size, the influencing range of every single graphene increased significantly. When the content of graphene remained the same, the total amount of graphene was inversely proportional to the size. For cases with low graphene size, the spatial distribution of graphene was relatively uniform. With the increase in graphene size, the non-uniformity of graphene distribution increased, and the extent of mutual contact and agglomeration of graphene also increased, as shown in Figure 3b,d,f. As the content of graphene increased from 1 wt.% to 4 wt.%, the spatial locations occupied by graphene increased rapidly and showed a dense distribution in the simulation area. It was worth noting that, in all simulated cases, a small number of blank areas without reinforcements could be observed, which was caused by the randomly generated algorithm of graphene.

**Figure 4:**
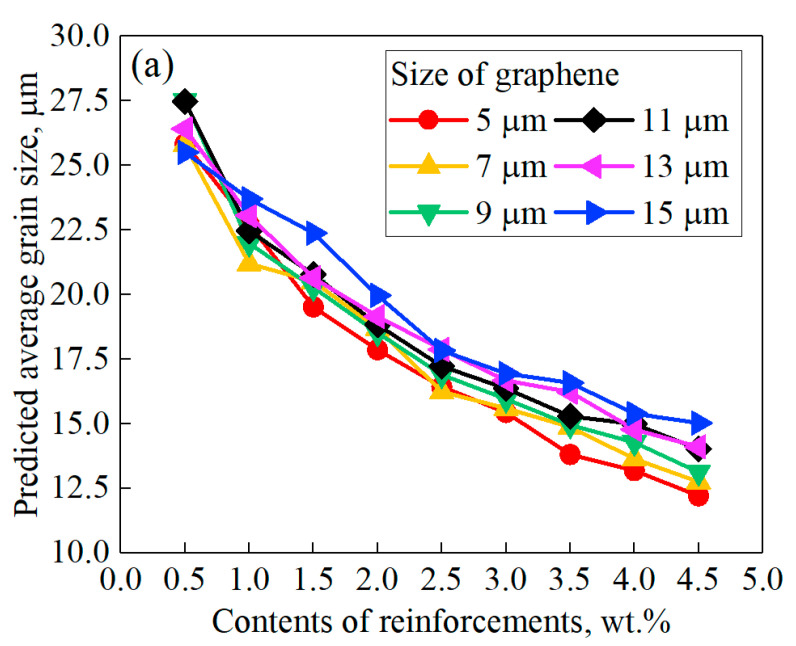

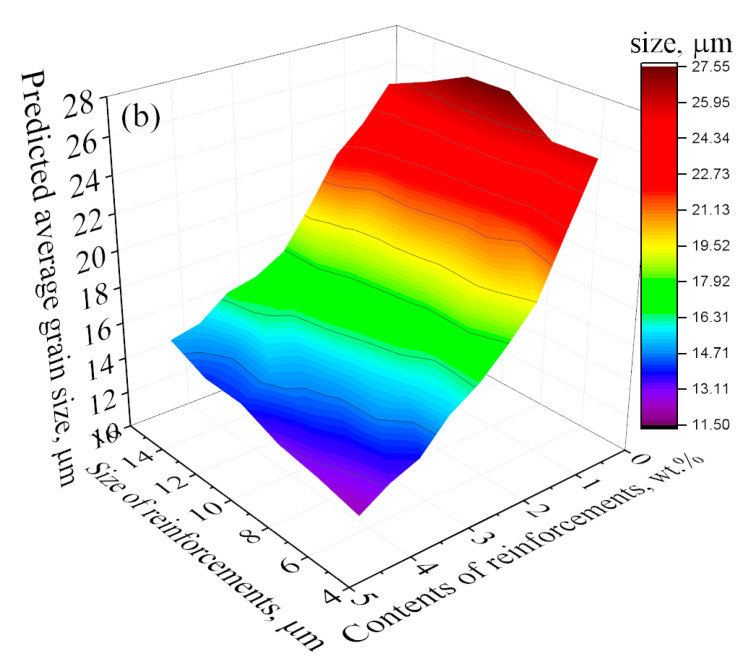
Predicted average grain sizes versus contents and sizes of reinforcements (**a**) curves (**b**) contour.

The predicted relationships between the final average grain size and parameters of graphene are shown in Figure 4. It can be seen that the average grain size decreased rapidly with the increase in graphene content, proving that graphene content is the main factor affecting the grain coarsening of GRAMCs. When the content of graphene was 0.5 wt.%, the final average grain size with different graphene sizes was 26.43 μm. However, when the content of graphene reached 4.5 wt.%, the final average grain size with different graphene sizes was 13.54 μm, which decreased by 48.77%. As can be seen from Figure 4a, the refining effect on grain size versus contents of graphene was not linearly distributed. When the contents of graphene were at relatively low values, such as 0.5 wt.% to 2.5 wt.%, the grain size decreased faster. When the contents of graphene continued to increase, such as more than 3 wt.%, the decline rate of grain size gradually slowed down. The predicted results showed that under the conditions of low mass ratio of graphene, the pinning effect of graphene on matrix grain size was more significant. However, with the continuous increase in graphene contents, the pinning effect was weakened, which showed a nonlinear regulation. This was due to the different grain growth rates under the conditions of low and high contents of graphene.

The predicted final grain size was also affected by the size of graphene, as shown in Figure 4b. The contour of predicted grain size showed that cases with smaller sizes of graphene tended to obtain a lower final grain size, which was more obvious when the contents of graphene were high. This was because under the same content condition, the increase in every single size of graphene reduced the total amount of graphene, thus reducing the pinning refinement effect on the whole region. Han et al. [21] reported the same regulations via simulation that decreasing the size of the particles reduced the final grain size of the matrix. SEM images of Ni-SiC nanocomposites from [29] also proved that grain size gradually decreased as reinforcing phases reduced in size. The predicted results were also consistent with the experimental observations of many graphene-reinforced composites. For example, grain size was observed to decrease with the decrease in graphene size in GNS-reinforced composite [30]. Hau et al. [31] observed an obvious decrease in grain size with the reduction in graphene size in Ni/GNPs nanocomposites. Size effects of graphene were also proven from the side that enhanced properties can be obtained with smaller graphene due to the strengthening mechanism of grain refinement [32].

**Figure 5:**
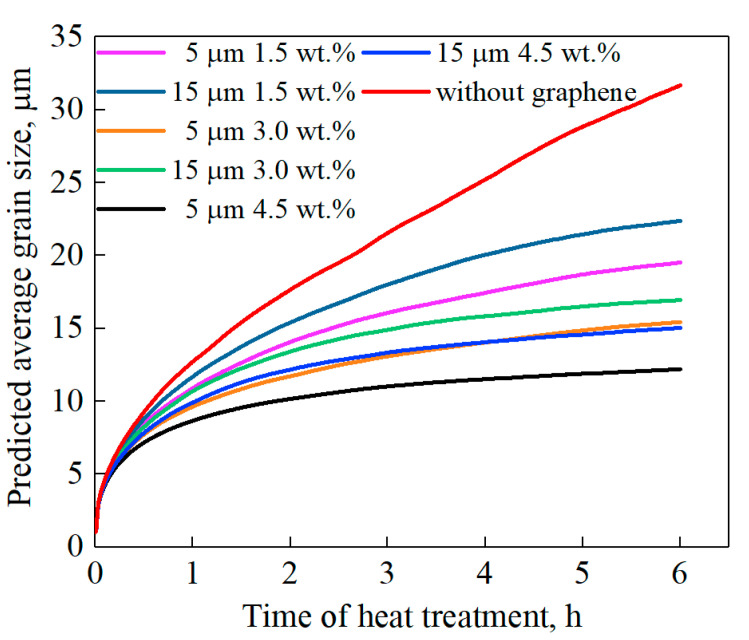
Predicted average grain size versus time of heat treatment.

In addition to the predicted final grain size, the variation of grain size versus time also confirmed the above regulations, as shown in Figure 5. For the case without graphene, the grain coarsening rate was relatively high, and still maintained a rapid increasing trend at the end of the simulation time. When graphene was introduced, the pinning refinement effect was reflected significantly. For all cases with graphene, the coarsening rates of grain size were all very fast at the beginning time of simulation, and then tended to decline and remain stable. Comparing cases with different graphene conditions, it can be seen that the content of graphene was still the most important factor to determine the extent of refinement. At the same time, when the contents of graphene are similar, grain refinement effect can be improved by reducing the size of graphene. For example, when the content and size of graphene was 4.5 wt.% and 15 μm, the trend of grain growth was quite similar with the case of 3 wt.% and 5 μm. The predicted final grain sizes were 15.03 μm and 15.44 μm, respectively. The grain growth curves of the two conditions were also well coinciding. The grain growth regulations predicted in this work were highly consistent with the growth curves of the pinning phenomenon reported by Agnoli et al. [33].

**Figure 6:**
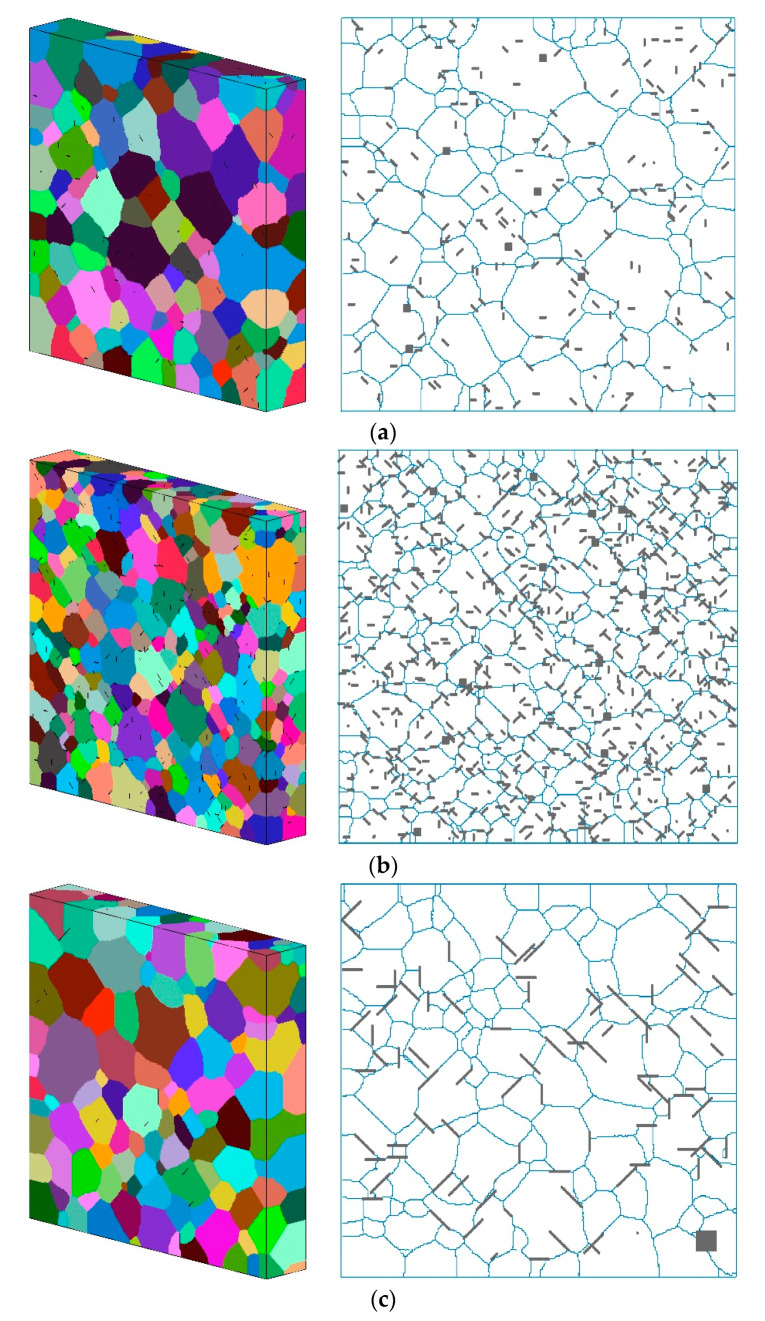

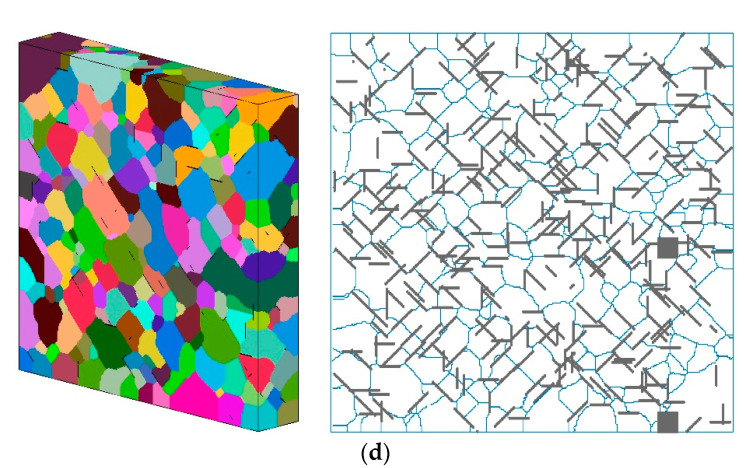
Predicted grain microstructure (**a**) 1 wt.% and 5 μm size of graphene, (**b**) 4.5 wt.% and 5 μm size of graphene, (**c**) 1 wt.% and 15 μm size of graphene, (**d**) 4.5 wt.% and 15 μm size of graphene.

The calculated results of the MCP model was a 300 × 300 × 50 matrix of grain orientation degrees. Graphene with orientation degree −1 was represented by gray points, cells with other orientation degrees were represented by random colors, and cells on grain boundaries were marked with blue lines. By this method, the predicted microstructure could be obtained, as shown in Figure 6. Four cases with two different sizes and contents of graphene were chosen for example. The 3D and 2D cross-sectional microstructure of the simulated region showed that most of the final grain morphology presented as hexagonal structures. Most of the graphene was distributed at the grain boundaries; however, some of them were located inside the grains. This can be verified by many experimental observation results of Al matrix composites [32,34,35]. When the content of graphene was low, the variation in grain size of the microstructure was relatively large. For example, as can be seen in Figure 6a,c, there were occasionally some grains with sizes much smaller than the average values at the boundaries or at the junction locations of several large grains. However, when the content of graphene was high, the overall grain size and morphology distribution were both more uniform. Comparing Figure 6b,d, it can be seen that when the content of graphene reached 4.5 wt.%, the final grain size was more refined under the condition of a smaller graphene size. Furthermore, it can also be seen in Figure 6d that in addition to the larger grain size, the graphene had more agglomeration and self-contacting or overlap phenomena at the grain boundaries. This is consistent with the findings reported by Azar et al. [36], who proved that agglomeration of graphene existed at grain boundaries when the content reached relatively high values by using transmission electron microscopic images. This might lead to defects of the obtained microstructure, and thus affect the mechanical properties of GRAMCs.

**Figure 7:**
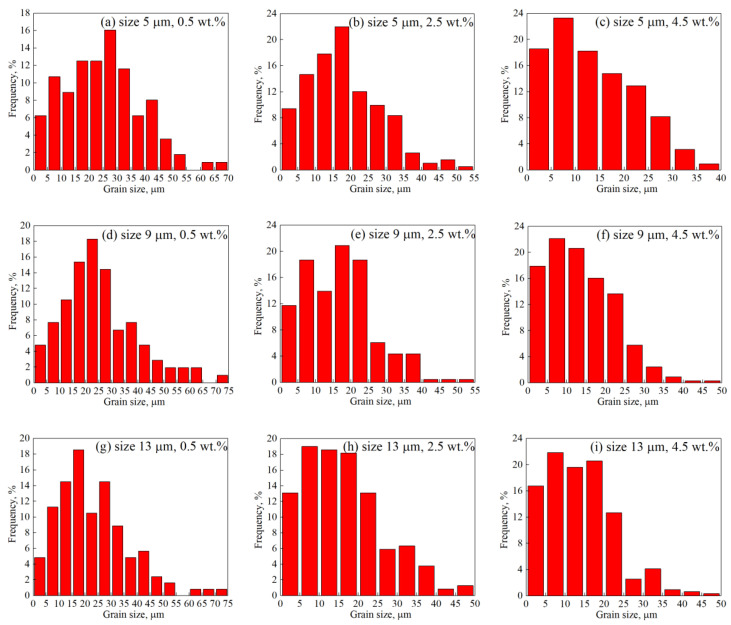
Frequency of grain size distribution under different conditions.

To investigate the uniformity of grain structures quantitatively, frequencies of grain size distribution were analyzed, as shown in Figure 7, with three different sizes and contents of graphene illustrated as examples. The statistical results showed that the content of graphene had a great impact on the grain size distribution. With the increase in content, the average and maximum grain sizes both decreased rapidly, as shown in Figure 7c,f,i. In the cases with low contents of graphene, a certain proportion of grains with large grain size appeared, which was due to the abnormal grain growth (AGG) phenomenon when the pinning effect was weak, as shown in Figure 7a,d,g, where it can be seen that a small proportion of grains with sizes ranging from 60 μm to 75 μm appeared. When the content of graphene was low, multiple peaks of the frequency of grain size were easy to be found. When the content of graphene became high, the frequency of grain size distribution was more stable.

The calculation standard deviation results of grain size showed that graphene contents had a great influence on the uniformity of grain size distribution, whereas graphene size had a much smaller influence. In Figure 7, the mean standard deviation of grain sizes of the three contents were 13.83 μm, 10.32 μm and 8.67 μm, respectively. This showed that under the condition of high contents, the proportion of abnormal grain growth decreased significantly and the grain size distribution was more uniform, which proves that the pinning effect induced by graphene can reduce the extents of AGG and heterogeneity. The statistical regulations of grain size distribution were also consistent with the previous grain morphology results.

## 4. Conclusions

In this work, an improved 3D Monte Carlo Potts model has been proposed to simulate the grain evolution of graphene-reinforced aluminum matrix composites (GRAMCs) during heat treatment for the first time. The modified 3D MCP model can predict spatial grain growth accurately with varying thermal conditions and various parameters of reinforcements. The model can be used for the quantitative design, simulation and preparation of GRAMCs. The prediction of grain growth has been carried out under various working conditions with graphene contents ranging from 0.5 wt.% to 4.5 wt.%, and sizes ranging from 5 μm to 15 μm. The corresponding thermal condition was 600 °C holding for 6 h. The following conclusions can be obtained:

1. Content of graphene has the most significant influence on the final grain size of GRAMCs, whereas the size of graphene can influence both the morphology of reinforcements and grain size. The average grain size decreased by 48.77% when the content increased from 0.5 wt.% to 4.5 wt.% for the simulated thermal condition;
2. High content of graphene leads to agglomeration and results in local defects or uneven grain morphology, which will reduce the mechanical properties. Increasing the size of graphene can reduce the total number of reinforcements and reduce the extent of agglomeration. However, larger graphene layers are more prone to self-contact and overlap;
3. The content of graphene can affect the uniformity of grain distribution after heat treatment, whereas the size of graphene has little influence. When the contents of reinforcements are low, abnormal grain growth (AGG) will occur, and the frequency of grain size distribution will show multi-peak phenomenon. With the increase in graphene content, the grain size distribution becomes more uniform and compact, and the extent of AGG decreases. Compared to the case with the lowest content of graphene, the standard deviation of grain size decreases by 37.31% when the content increases to 4.5 wt.%.

## Figures and Tables

**Table 1:** Parameters and constants in the model.

Parameter	Value
Average number per unit area, *Z*	4.31 × 10^20^ m^−2^
Planck’s constant, *h*	6.624 × 10^−34^ J·s
Accommodation probability, *A*	1.0
Gas constant, *R*	8.31 J·mol^−1^·K^−1^
Avogadro’s number, *N*_a_	6.02 × 10^23^·mol^−1^
Atom molar volume, *V*_m_	1.0 × 10^−5^ m^3^·mol^−1^
Fusion entropy, ∆*S*_f_	11.5 J·mol^−1^·K^−1^
Boundary energy, *γ*	0.5 J·m^−2^
Activation enthalpy, *Q*	146 kJ·mol^−1^
